# Barriers and incentives influencing the use of partograph in Nigeria: A comprehensive review

**DOI:** 10.1097/MD.0000000000038389

**Published:** 2024-05-31

**Authors:** Chukwuka Elendu, George Davidson, John N. Wali, Godstime U. Sampson, Ucheawaji S. Eneyo, Philip E. Ebosie, Paschal C. Opara, Prince N. Davidson, Darlington U. Davidson, Junior Davidson

**Affiliations:** a University of California, Santa Cruz, Santa Cruz, CA; b University of Port Harcourt, Choba, Nigeria; c University of Chester, Chester, United Kingdom; d Peoples’ Friendship University, Moscow, Russia; e Imo State University, Owerri, Nigeria; f Abia State University, Uturu, Nigeria.

**Keywords:** ethical considerations, global best practices, implementation strategies, maternal healthcare, obstetric care, partograph, sustainability

## Abstract

This comprehensive review examines the barriers and incentives influencing the use of partographs in maternal healthcare within Nigeria. Maternal mortality remains a critical concern in the country, making it imperative to evaluate the factors that shape the adoption of essential tools like the partograph. The literature review underscores the global significance of partograph utilization, emphasizing its role in improving maternal outcomes. A particular focus is placed on existing studies and findings relevant to Nigeria, providing a nuanced understanding of the challenges and opportunities faced by healthcare providers. The article delves into the barriers hindering the widespread adoption of partographs in Nigeria, including issues related to training, resource availability, and cultural considerations. Additionally, it explores the incentives that can positively influence healthcare practitioners and facilities to integrate partographs into their maternal care protocols. Government policies and initiatives in Nigeria related to maternal healthcare and partograph use are also analyzed, shedding light on the broader contextual factors impacting implementation. Through examining case studies, the review presents real-world examples that illustrate successful and challenging instances of partograph implementation. The article concludes with actionable recommendations to overcome identified barriers and enhance incentives for effectively integrating partographs into maternal healthcare practices in Nigeria. This study contributes valuable insights to the ongoing discourse on improving maternal healthcare, emphasizing the need for tailored strategies in the Nigerian context.

## 1. Introduction

Maternal healthcare is a global imperative, and within the context of Nigeria, where maternal mortality remains a significant public health concern, the need for effective tools and protocols is paramount. The partograph, a graphical representation of a woman’s labor progress, is vital in monitoring and managing maternal well-being during childbirth.^[1]^ This paper undertakes a comprehensive review to explore the barriers and incentives that shape the utilization of partographs within the Nigerian healthcare landscape.

Globally recognized for its potential to improve maternal outcomes, the partograph has been integral to evidence-based practices in obstetrics.^[1]^ However, its adoption in Nigeria faces multifaceted challenges, ranging from healthcare provider training to resource limitations and cultural considerations.^[2]^ Understanding these barriers is crucial in developing targeted strategies to enhance the integration of the partograph into routine maternal care.

Conversely, various factors incentivize healthcare practitioners and facilities to embrace the partograph. These incentives, be they policy-driven or from successful implementation models, play a pivotal role in fostering a positive environment for the consistent use of partographs in maternal healthcare.^[3]^ By examining barriers and incentives, this study aims to provide nuanced insights into the dynamics surrounding partograph utilization in Nigeria.

Moreover, the review includes a meticulous exploration of existing literature on partograph usage globally, with a specific focus on studies and findings pertinent to Nigeria. This contextual analysis allows us to draw connections between global best practices and the unique challenges faced within the Nigerian healthcare system.^[4]^

In tandem with analyzing government policies and initiatives related to maternal healthcare in Nigeria, this study seeks to contribute evidence-based recommendations for overcoming barriers and optimizing incentives. Through the lens of case studies, both domestic and international, we aim to illustrate instances of successful integration as well as challenges faced in incorporating the partograph into routine maternal care protocols.^[5]^

By addressing these aspects comprehensively, this review adds to the knowledge on maternal healthcare in Nigeria. It provides practical insights for policymakers, healthcare professionals, and researchers working towards improved maternal outcomes.

## 2. Literature review

The global significance of partograph utilization in maternal healthcare has been extensively documented, highlighting its role as a crucial tool in monitoring labor progress and enhancing maternal outcomes.^[1]^ Within this context, Nigeria grapples with a complex interplay of factors that influence the adoption and integration of partographs into routine clinical practice.

Studies worldwide consistently emphasize the positive impact of partographs on reducing maternal morbidity and mortality.^[1]^ This graphical representation of labor progress allows healthcare providers to make timely interventions, ensuring safer deliveries and better maternal health outcomes. However, despite these benefits, adopting partographs in Nigeria faces notable challenges.^[6]^

Healthcare provider training stands out as a critical barrier. Limited access to comprehensive training programs on partograph usage contributes to a gap in knowledge and skills among practitioners.^[2]^ This training deficiency hampers the tool’s effective implementation, impacting its potential benefits on maternal health.

Resource limitations within the Nigerian healthcare system also pose substantial barriers to partograph utilization.^[3]^ More availability of essential materials, such as paper forms and other monitoring equipment, is necessary to ensure the consistent use of partographs across healthcare facilities.^[7]^ Addressing these resource challenges is pivotal for ensuring the sustained integration of partographs into routine maternal care.

Cultural considerations further complicate the landscape of partograph adoption in Nigeria.^[4]^ Cultural beliefs and practices surrounding childbirth may influence healthcare-seeking behavior and acceptance of medical interventions.^[8]^ Understanding and addressing these cultural nuances are essential for tailoring interventions that resonate with local communities and foster the acceptance of partographs in maternal care.

In contrast to these barriers, various incentives can positively influence the uptake of partographs in Nigeria. Government policies and initiatives play a pivotal role in shaping the healthcare landscape. Policies prioritizing maternal health and advocating for standardized partograph usage can catalyze change.^[5]^

Successful implementation models within Nigeria and globally offer valuable insights into effective strategies for integrating partographs into routine maternal care.^[6]^ These case studies highlight the importance of multifaceted approaches, including training programs, resource allocation, and community engagement.^[9]^

## 3. Methodology

The methodology employed in this article involves a comprehensive review and synthesis of existing literature on the utilization of partographs in maternal healthcare, with a primary focus on the context of Nigeria. No original data collection, including Focus Group Discussions, questionnaires, or expert interviews, was conducted for this review.

*Literature search*: A systematic literature search was conducted using reputable databases such as PubMed, ScienceDirect, and Google Scholar. The search included articles, reviews, and reports published between January 1, 2023, and December 31, 2023, explicitly emphasizing studies on partograph utilization in maternal healthcare in Nigeria.

*Inclusion and exclusion criteria*: Articles were included based on their relevance to using partographs, barriers, and incentives in maternal healthcare, focusing on the Nigerian context. Exclusion criteria encompassed studies unrelated to partographs, those not specific to the Nigerian setting, and articles lacking relevance to barriers or incentives.

*Data extraction and synthesis*: Data from selected studies were extracted, focusing on key findings related to barriers and incentives in using partographs. The synthesized information was organized thematically to provide a structured and comprehensive overview.

*Quality assessment*: A quality assessment of selected studies was conducted to ensure the inclusion of high-quality and relevant literature. The evaluation considered study design, sample size, methodology, and the rigor of data analysis.

*Ethical considerations*: As this review solely relies on existing literature, ethical considerations related to primary data collection, such as informed consent and confidentiality, were not applicable. All information presented is based on publicly available and previously published studies.

## 4. Discussion

### 4.1. Historical context of partograph utilization in Nigeria

The historical evolution of partograph utilization in Nigeria is a captivating journey marked by dynamic shifts in maternal healthcare practices. Rooted in a timeline spanning several decades, this narrative unveils the intricate interplay of historical, cultural, and healthcare factors that have shaped the trajectory of partograph integration.^[10]^

In the early stages, the utilization of partographs was introduced as a response to the escalating rates of maternal morbidity and mortality. Pioneering efforts by healthcare practitioners sought innovative tools to enhance the monitoring of labor progression and early detection of complications. The introduction of the partograph, credited to visionary healthcare leaders, aimed to revolutionize obstetric care, and reduce adverse outcomes during childbirth.^[11]^

During the 1970s and 1980s, Nigeria witnessed a gradual but significant adoption of the partograph within select healthcare facilities. The pioneering initiatives, often spearheaded by dedicated maternal health advocates and educators, emphasized the importance of standardized labor monitoring. While initial implementation faced challenges, the persistence of these pioneers laid the groundwork for broader acceptance and utilization.^[12]^

The 1990s marked a turning point with increased recognition of the partograph’s efficacy in preventing prolonged labor and reducing maternal and neonatal complications. This era saw a surge in training programs for healthcare providers, emphasizing the correct interpretation and application of the partograph. Government-led initiatives and collaborations with international organizations played a pivotal role in scaling training efforts, contributing to a more widespread tool integration.^[13]^

As the new millennium unfolded, technological advancements brought about electronic partographs, adding a layer of sophistication to labor monitoring. These digital tools facilitated real-time data recording, enhanced decision support, and improved the accessibility of maternal health information. This era witnessed a blend of traditional paper-based partographs and emerging electronic solutions, highlighting Nigeria’s adaptability to maternal healthcare practices.^[14]^

The historical context has evolved in recent years to include a growing emphasis on community engagement and awareness. Advocacy campaigns led by governmental and nongovernmental entities have aimed to demystify the partograph, fostering a culture of acceptance and understanding within local communities. This grassroots approach recognizes the significance of community involvement in ensuring the sustained utilization of the partograph.^[15]^ Nigeria, as a critical player in maternal healthcare within the African continent, grapples with challenges and opportunities in the widespread adoption of partographs. The partograph, a graphical tool for monitoring labor, has the potential to enhance maternal and neonatal outcomes significantly. Table 1 delves into the landscape of partograph utilization in Nigeria, considering various factors that shape its implementation.

**Table 1:** Overview of partograph utilization in Nigeria.

Factors	Description
Healthcare infrastructure challenges	Nigeria faces infrastructural challenges, including uneven distribution of healthcare facilities and limited access to essential resources.
Cultural influences on maternal healthcare	Cultural norms and traditional birthing practices in Nigeria influence maternal healthcare decisions and may impact the acceptance of partographs.
Healthcare workforce capacity	The capacity of the healthcare workforce in Nigeria is crucial. Ongoing training programs and initiatives are essential to enhance proficiency in partograph utilization.
Government initiatives and policies	Government policies and initiatives play a pivotal role. Analyzing existing programs related to maternal healthcare and partograph utilization is vital.
Data management and technology integration	Assessing the readiness of Nigerian healthcare facilities to adopt electronic partographs and exploring potential barriers to technological integration is imperative.
Community engagement programs	Community-based programs aim to raise awareness about maternal healthcare, including using partographs. Successful initiatives engage communities and encourage acceptance.
Collaborations with NGOs	Partnerships between the Nigerian government and NGOs on maternal healthcare significantly improve outcomes and promote partograph utilization.
Economic considerations	The economic aspects of maternal healthcare in Nigeria, including affordability, insurance coverage, and potential economic barriers, are critical considerations in partograph utilization.

As Nigeria navigates the complex landscape of maternal healthcare, understanding the multifaceted aspects of partograph utilization is essential. The challenges and successes in incorporating this tool into routine practices contribute to shaping maternal healthcare outcomes nationwide.

The historical journey of partograph utilization in Nigeria is a testament to the resilience of healthcare professionals, policymakers, and advocates committed to improving maternal outcomes.^[16]^ From its inception as a groundbreaking tool to contemporary efforts to bridge healthcare disparities, the historical context of partograph utilization reflects a narrative of progress, challenges, and a collective commitment to advancing maternal healthcare in Nigeria.

### 4.2. Barriers to partograph use in Nigeria

Navigating the landscape of maternal healthcare in Nigeria reveals a tapestry woven with intricate challenges that have contributed to barriers in effectively utilizing partographs. This narrative peels back the layers, delving into the multifaceted dimensions that hinder the seamless integration of this crucial tool.

One significant barrier lies in the realm of healthcare infrastructure. Like many developing nations, Nigeria needs to improve its infrastructure, directly impacting the consistent implementation of partographs. Insufficient access to essential resources, including paper-based partograph forms and monitoring equipment, hampers healthcare providers’ ability to consistently employ this tool across various healthcare settings.^[17]^

Human resource constraints emerge as a formidable hurdle, with healthcare facilities facing staffing levels and workforce capacity challenges. The need for more skilled healthcare providers and high patient-to-provider ratios strains the ability to allocate dedicated attention to each laboring woman.^[18]^ This scarcity of resources contributes to a situation where the meticulous and time-sensitive monitoring required by the partograph may be compromised.^[2]^

Resistance to change within healthcare systems and among providers constitutes another substantial barrier. Integrating new practices, such as the routine use of partographs, often encounters skepticism and reluctance rooted in established routines and apprehensions about the perceived complexities of implementation. Overcoming this resistance necessitates targeted training and change management strategies to instill confidence and foster a positive shift in mindset.^[19]^

Socio-cultural factors play a nuanced role in influencing the acceptance and adherence to partograph usage. Deeply ingrained cultural norms, beliefs, and practices surrounding childbirth may diverge from the structured approach advocated by the partograph. Bridging this cultural gap requires sensitivity to local contexts, community engagement, and recognizing culture’s role in shaping maternal healthcare practices.^[14]^

The limitations of healthcare education and training programs contribute to barriers to partograph use. Inadequate emphasis on maternal health protocols, including partograph interpretation, during formal healthcare education programs results in a gap in the knowledge and skills of healthcare providers.^[20]^ Continuous professional development opportunities are essential to address these gaps and ensure a competent workforce.^[5]^

Data management and record-keeping challenges further compound the barriers to partograph use. In settings where paper-based systems prevail, misplaced or incomplete records hinder the accurate assessment of labor progression. While promising, the transition to electronic health records introduces challenges related to technology literacy and infrastructure.^[21]^

Economic considerations present yet another layer of complexity. Limited financial resources within healthcare systems may hinder investments in technology upgrades, infrastructure improvements, and comprehensive training programs. The upfront costs associated with partograph implementation may be perceived as prohibitive, requiring a strategic approach to justify the long-term benefits.^[17]^

In navigating these intricate barriers, it becomes evident that addressing each Challenge demands a holistic and context-specific approach. The path toward overcoming barriers to partograph use in Nigeria necessitates collaborative efforts, encompassing policy reforms, targeted training initiatives, community engagement, and strategic investments in healthcare infrastructure and education.^[22]^

### 4.3. Incentives for partograph use

Incentivizing the utilization of partographs in the landscape of maternal healthcare in Nigeria is an intricate dance between acknowledging existing challenges and nurturing positive shifts in practice. This narrative unravels the diverse incentives that hold the potential to transform the adoption and consistent application of partographs, creating a ripple effect for improved maternal and neonatal outcomes.^[23]^

At the forefront of these incentives lies the promise of enhanced communication and decision-making among healthcare providers. The partograph becomes a shared language when embraced as a standard tool, facilitating a more cohesive and informed approach to labor monitoring. This interconnectedness among healthcare teams fosters a collaborative environment where timely interventions can be coordinated, ultimately reducing the risk of adverse maternal and neonatal events.^[1]^

Improved risk assessment emerges as a compelling incentive, underlining the potential of partographs to identify complications in the early stages of labor. By visualizing labor progression, healthcare providers can proactively assess and address emerging risks, preventing the escalation of complications. This proactive stance contributes to better maternal outcomes and instills confidence in healthcare providers regarding the efficacy of partograph utilization.^[24]^

Partograph utilization brings forth the incentive of streamlined documentation and record-keeping. In an era where the accuracy and accessibility of patient records are paramount, the standardized format of partographs offers a tangible advantage. When consistently maintained, the visual representation of labor progression is a valuable historical record that can inform subsequent pregnancies and contribute to evidence-based decision-making.^[23]^

A key incentive for partograph use lies in its potential to empower healthcare providers with a sense of control and mastery over the labor monitoring process. As providers witness the positive impact of timely interventions facilitated by partographs, a sense of professional accomplishment is cultivated. This intrinsic motivation and a heightened awareness of the positive outcomes associated with partograph utilization drive sustained adherence to best practices.^[24]^

Community engagement emerges as a pivotal incentive, positioning partograph use as a symbol of improved maternal care within local contexts. Advocacy efforts that demystify the partograph and engage communities in understanding its significance contribute to a cultural shift. When communities actively embrace the tool, expectant mothers advocate for its use, further strengthening the incentive structure and creating a demand for improved maternal healthcare practices.^[25]^

Recognition and acknowledgments form external incentives that resonate with healthcare providers. Acknowledgments of adherence to partograph utilization guidelines through awards, professional recognition, or continuous education opportunities serves as both a motivator and a reinforcement mechanism. This external validation reinforces the importance of partograph utilization in the broader context of maternal healthcare best practices.^[26]^

While secondary to the intrinsic and professional motivators, financial incentives play a role in sustaining partograph utilization. Healthcare systems prioritizing the availability of necessary resources, including partograph forms and monitoring equipment, demonstrate a commitment to facilitating the consistent use of this tool. Investments in technology upgrades and training programs contribute to the financial backbone supporting partograph implementation.^[27]^

In conclusion, the incentives for partograph are used to weave a narrative of empowerment, collaboration, and positive outcomes. By addressing challenges through a strategic alignment of intrinsic, community-driven, and external motivators, the adoption and sustained use of partographs can be nurtured. This journey promises to transform labor monitoring practices in Nigeria and serve as a beacon for improved maternal healthcare globally.

### 4.4. Government policies and initiatives

The landscape of partograph utilization in Nigeria is intricately woven into the fabric of government policies and initiatives that address the complex challenges of maternal healthcare. This narrative explores the historical journey of policy interventions, their impact on partograph implementation, and the evolving role of the Nigerian government in shaping the trajectory of maternal health practices.

In the early stages, government policies laid the foundation for recognizing the significance of standardized labor monitoring. The introduction of guidelines advocating for using partographs within healthcare facilities signaled a pivotal shift in maternal healthcare protocols. These early policy initiatives aimed to establish a unified approach to labor monitoring, acknowledging the potential of partographs to improve outcomes.^[28]^

As the timeline progressed, the Nigerian government embarked on initiatives to enhance healthcare infrastructure, recognizing its pivotal role in supporting partograph utilization. Investments in the procurement of monitoring equipment, training programs for healthcare providers, and distributing standardized partograph forms signaled a commitment to overcoming infrastructure-related barriers. These initiatives sought to create an enabling environment conducive to the consistent use of partographs across diverse healthcare settings.^[29]^

The evolution of government policies witnessed strategic collaborations with international organizations and nongovernmental entities. Joint initiatives focused on capacity-building, training-of-trainers programs, and disseminating best practices in partograph utilization. These collaborative efforts enriched the skill set of healthcare providers and contributed to developing context-specific guidelines aligned with global best practices.^[30]^

A notable shift has occurred in recent years towards integrating digital health solutions within government policies. The embrace of electronic partographs reflects a commitment to technological advancements that can streamline data management, enhance decision support, and improve overall maternal healthcare outcomes. Government-backed initiatives in technology integration signify a recognition of innovation’s role in overcoming barriers to partograph utilization.^[31]^

Policy initiatives prioritizing community engagement and awareness have also shaped the landscape of partograph utilization. Government-led advocacy campaigns, often collaborating with nongovernmental organizations, aim to demystify the partograph and empower local communities. These initiatives position the partograph not just as a clinical tool but as a symbol of improved maternal care that resonates within the cultural context of Nigerian communities.^[32]^

Government policies and initiatives have increasingly focused on fostering a culture of continuous quality improvement (CQI) within maternal healthcare. Integrating CQI principles emphasizes ongoing assessments, feedback loops, and data-driven decision-making. This iterative approach aligns with the dynamic nature of partograph utilization, allowing for real-time adjustments and improvements in response to emerging challenges.^[33]^

In conclusion, the narrative of government policies and initiatives reflects a dynamic journey of progress, challenges, and strategic interventions. From the early recognition of partographs in maternal healthcare protocols to contemporary efforts embracing digital solutions and community-driven initiatives, the Nigerian government plays a pivotal role in shaping the landscape of partograph utilization. This ongoing commitment holds the potential to transform maternal healthcare practices further, ensuring that the benefits of partograph utilization are realized at every healthcare system level.

## 5. Case studies

Embarking through compelling case studies illuminates the nuanced landscape of partograph utilization in maternal healthcare in Nigeria. These narratives encapsulate the real-world challenges, successes, and transformative impacts that unfold within diverse healthcare settings, offering a vivid tapestry of experiences.

### 5.1. Case study 1: a rural health facility in northern Nigeria

In the heart of a rural community, where access to healthcare resources is limited, a dedicated team of healthcare providers embraced partograph utilization as a catalyst for change. With the support of a government-led initiative, training programs were conducted to empower local midwives and nurses. Introducing simple yet effective partograph forms tailored to the community’s context facilitated a paradigm shift in labor monitoring. Over time, this grassroots initiative led to a notable reduction in prolonged labor instances, highlighting the adaptability of partographs in resource-limited environments.

### 5.2. Case study 2: urban maternity hospital in Lagos

Navigating the bustling urban landscape of Lagos, a maternity hospital integrated electronic partographs into its routine practices. Leveraging technological advancements, healthcare providers seamlessly transitioned from traditional paper-based records to digital solutions. The electronic partograph streamlined data management and facilitated real-time decision support. The hospital’s commitment to innovation underscored the transformative potential of digital tools in enhancing the accuracy and efficiency of labor monitoring.

### 5.3. Case study 3: community-led advocacy in southeastern Nigeria

In a vibrant community in southeastern Nigeria, community leaders and healthcare advocates joined forces to demystify the partograph. Expectant mothers became champions for partograph utilization through engaging workshops, awareness campaigns, and community dialogs. The cultural acceptance and community-driven approach transformed the partograph from a clinical tool to a symbol of improved maternal care. This grassroots movement illustrated the profound impact of community engagement in fostering a culture of maternal health awareness.

### 5.4. Case study 4: teaching hospital in Abuja

Comprehensive training programs were implemented within the bustling corridors of a teaching hospital in the capital city to equip healthcare professionals with advanced partograph interpretation skills—the hospital, serving as a hub for medical education, prioritized continuous professional development. The integration of case-based learning and simulation exercises enhanced the competency of healthcare providers in using partographs as dynamic tools for risk assessment and intervention planning.

### 5.5. Case study 5: nongovernmental organization (NGO) initiative in the Niger Delta

In the remote communities nestled along the Niger Delta, an NGO undertook a holistic initiative to address barriers to partograph utilization. Beyond providing training and resources, the NGO collaborated with local leaders to overcome resistance to change. By aligning partograph utilization with existing cultural practices and incorporating traditional birth attendants into the training programs, the initiative showcased the importance of culturally sensitive approaches in enhancing the acceptance and utilization of partographs.

These case studies underscore the diversity of challenges and successes in partograph utilization across varied settings in Nigeria. They serve as illuminating vignettes, highlighting the adaptability, resilience, and transformative potential of partographs in the rich tapestry of maternal healthcare experiences.

## 6. Global best practices in partograph implementation

As we traverse the global landscape of maternal healthcare, insights from diverse settings unveil a tapestry of best practices in partograph implementation that transcend borders. These practices, drawn from successful initiatives worldwide, offer a nuanced understanding of effective strategies that have enhanced the utilization of partographs.

Sweden’s collaborative care and interdisciplinary communication model has emerged as a beacon of best practice.^[34]^ Integrating partographs within a comprehensive electronic health record system facilitates seamless communication among healthcare providers, ensuring a unified approach to labor monitoring. This model emphasizes the importance of standardized protocols and technological integration in optimizing the impact of partographs on maternal outcomes.^[1]^

In Ghana, a community-centered approach has demonstrated significant success in promoting partograph utilization. A sense of ownership and cultural acceptance has been fostered by involving traditional birth attendants and local community leaders in training programs. This model recognizes the importance of engaging with communities to dispel myths and misconceptions surrounding partographs, creating a supportive environment for their use.^[2]^

The experience in Australia highlights the transformative potential of CQI frameworks. By embedding partograph utilization within a broader quality improvement strategy, healthcare facilities have established feedback loops, regular audits, and ongoing training programs. This iterative approach ensures that partograph practices evolve in response to emerging challenges and opportunities for improvement.^[33]^

In Japan, a focus on standardized education and certification programs has contributed to successfully integrating partographs into routine maternal healthcare practices. Comprehensive training modules and certification requirements for healthcare providers underscore the commitment to ensuring a competent and skilled workforce. This approach recognizes the critical role of education in overcoming barriers to partograph utilization.^[4]^

The landscape in South Africa showcases the impact of policy integration in driving best practices. Government-led initiatives that mandate partographs within national maternal health guidelines have created a standardized approach across healthcare facilities. This top-down approach ensures consistency, accountability, and a shared commitment to incorporating partographs as essential tools for labor monitoring.^[35]^

In the United States, the advent of telehealth has extended its influence on partograph utilization. Remote monitoring and virtual consultations have been integrated into maternal healthcare, allowing healthcare providers to assess partographs and offer guidance from a distance. This approach not only enhances accessibility but also demonstrates the potential for leveraging technology to overcome geographical barriers.^[36]^

The global best practices in partograph implementation converge on common themes—collaboration, community engagement, continuous improvement, education, policy integration, and technological innovation. These insights, gleaned from diverse healthcare systems, underscore the importance of context-specific strategies that align with local needs and challenges. By weaving together these global threads of best practices, the international community can foster a collective commitment to advancing maternal healthcare outcomes by effectively utilizing partographs.

## 7. Healthcare infrastructure and technology integration in partograph utilization

The intersection of healthcare infrastructure and technology integration presents a dynamic landscape in partograph utilization. From robust healthcare systems in developed nations to resource-constrained settings, the interplay between infrastructure development and technology adoption shapes the efficacy of partographs in maternal healthcare.

In high-income countries like Norway, seamless technology integration within well-established healthcare infrastructures has redefined partograph utilization. Electronic health records (EHRs) synchronize with partograph data, offering real-time insights to healthcare providers. This interoperability enhances decision support, streamlines communication, and establishes a comprehensive framework for maternal healthcare within the broader healthcare infrastructure.^[1]^

Conversely, innovative approaches harness technology to bridge infrastructural gaps in resource-constrained regions such as rural India. Mobile health (mHealth) applications designed for low-resource settings facilitate partograph utilization even in areas with limited access to traditional healthcare infrastructure. This decentralized model leverages smartphones to empower frontline healthcare workers, ensuring that partographs become integral tools despite infrastructural challenges.^[2]^

In China, a hybrid model of technology integration unfolds within a rapidly advancing healthcare infrastructure. Urban maternity hospitals embrace electronic partographs, seamlessly integrated with advanced monitoring equipment. Simultaneously, remote rural clinics leverage telehealth platforms to connect with central databases, enabling the review, and consultation of partographs by specialists. This dual approach exemplifies the adaptability of technology to diverse infrastructural contexts within a single healthcare system.^[37]^

A unique narrative emerges from Nigeria, where the digital divide challenges nationwide technology integration. Urban teaching hospitals showcase advancements in partograph integration within electronic health systems, yet rural clinics face infrastructure limitations. Government-led initiatives aim to address this disparity by investing in technology and infrastructure, recognizing the need for comprehensive development to maximize the impact of partographs across diverse settings.^[4]^

The role of artificial intelligence (AI) unfolds as a transformative force within healthcare infrastructures globally. In countries like the United States, AI algorithms analyze partograph data to predict labor complications, offering a proactive approach to intervention. Integrating AI into existing healthcare infrastructures reflects a commitment to harnessing cutting-edge technology to enhance the precision and efficiency of partograph utilization.^[5]^

The evolving landscape of healthcare infrastructure and technology integration in partograph utilization underscores the importance of context-specific strategies. Whether navigating well-established systems or overcoming infrastructural challenges, the adaptability of technology becomes a key determinant in realizing the full potential of partographs. As the global healthcare community continues to embrace technological innovations, the synergy between infrastructure development and technology adoption will play a pivotal role in shaping the future of maternal healthcare.

## 8. Community perspectives on maternal healthcare and partograph utilization

The tapestry of maternal healthcare and partograph utilization is intricately woven with the vibrant threads of community perspectives, offering a rich narrative that extends beyond clinical settings. These perspectives, shaped by cultural norms, beliefs, and lived experiences, contribute profoundly to the reception and integration of partographs within diverse communities.

In rural Kenya, where community ties run deep, accepting partographs is intertwined with communal practices surrounding childbirth. Traditional birth attendants, revered figures within the community, are pivotal in bridging the gap between cultural expectations and modern healthcare practices. By incorporating partographs into their repertoire, these attendants serve as cultural mediators, fostering a sense of trust and familiarity with maternal healthcare interventions.^[1]^

Conversely, in urban communities in Brazil, the intersection of cultural perspectives and socioeconomic dynamics paints a nuanced picture of partograph utilization. Here, community members often engage in dialogs with healthcare providers, expressing concerns about the unfamiliarity of the partograph. Collaborative initiatives involving community leaders in awareness campaigns and educational programs demystify the tool, fostering a sense of partnership in maternal healthcare decision-making.^[2]^

The landscape in rural Nigeria reveals deeply rooted beliefs surrounding childbirth and maternal health. Elders and community leaders, revered for their wisdom, influence the community’s perception of healthcare interventions. Embracing a community-driven approach, healthcare providers engage in open dialogs, acknowledging cultural practices while introducing the benefits of partograph utilization. This coalescence of cultural sensitivity and healthcare awareness paves the way for a more harmonious integration of partographs within local contexts.^[38]^

In the highlands of Peru, where indigenous communities uphold centuries-old traditions, community perspectives on maternal healthcare weave a narrative of resilience and adaptation. Indigenous midwives, revered for their traditional knowledge, become conduits for introducing partographs. By aligning the tool with cultural values and emphasizing its role in preserving the well-being of both mothers and infants, partographs become embraced as a harmonious blend of modern healthcare practices and cultural heritage.^[4]^

Community-driven initiatives in urban India showcase the power of grassroots movements in shaping perspectives on partograph utilization. Advocacy groups, often led by women from within the community, engage in storytelling sessions and peer-to-peer education. The narratives shared highlight personal experiences with partographs, dismantling myths and instilling a sense of empowerment within the community. This participatory approach fosters a positive shift in community perspectives, positioning partographs as valuable tools in ensuring safe maternal outcomes.^[5]^

These diverse community perspectives underscore the importance of recognizing the intricate interplay between cultural contexts and maternal healthcare practices. From traditional birth attendants acting as cultural mediators to community-led initiatives fostering dialogue and empowerment, integrating partographs within communities is a dynamic and evolving journey. Healthcare providers can forge meaningful collaborations by embracing these perspectives, ensuring that partograph utilization resonates with the communities’ values and aspirations.

## 9. Challenges in scaling successful models of partograph utilization

As we delve into the complexities of scaling successful models of partograph utilization, a myriad of challenges surfaces, underscoring the intricacies involved in extending effective practices beyond initial implementations. The narratives from diverse settings provide insights into the hurdles faced when attempting to replicate and expand successful partograph programs.

In a bustling urban hospital in Bangladesh, where a pilot project demonstrated significant improvements in maternal outcomes through partograph utilization, the Challenge of workforce scalability became apparent. The success achieved by a dedicated team of healthcare providers was contingent on their expertise and commitment. Expanding this model to other healthcare facilities necessitated comprehensive training programs, highlighting the need for a scalable and sustainable approach to building a skilled workforce across diverse settings.^[1]^

Similarly, in a rural community in Tanzania, where partograph utilization positively impacted maternal health, the Challenge of resource limitations emerged as a significant barrier to scaling. The initial success relied on the availability of essential resources, such as partograph forms, monitoring equipment, and skilled personnel. Replicating this success in resource-constrained settings required strategic investments in infrastructure, technology, and ongoing support to ensure sustained partograph utilization.^[2]^

The digital literacy challenge became evident in an innovative mHealth initiative in Peru, which demonstrated the adaptability of partographs to remote settings. While the initial pilot engaged community health workers proficient in mobile technology, scaling to other regions faced obstacles related to varying levels of digital literacy among healthcare providers. Overcoming this challenge demanded tailored training programs and continuous support to ensure the effective integration of digital partographs across diverse contexts.^[39]^

The landscape of scaling successful partograph models in a diverse region of Nigeria brought attention to the Challenge of cultural sensitivity. The initial success hinged on community engagement strategies that resonated with local cultural norms and beliefs. Expanding this model to other communities required a nuanced understanding of diverse cultural contexts, necessitating customized approaches that respect and integrate local perspectives on maternal healthcare and childbirth practices.^[40]^

In the context of a national policy initiative in South Africa that showcased the successful integration of partographs into routine maternal health protocols, the challenge of standardization emerged. While the policy framework provided a unified approach, adapting to diverse healthcare settings highlighted the importance of flexibility within standardized guidelines. Tailoring implementation strategies to accommodate variations in infrastructure, resources, and community dynamics proved essential in achieving widespread success.^[5]^

The journey of scaling successful models of partograph utilization reveals that replicating success is not a one-size-fits-all endeavor. Workforce capacity, resource availability, digital literacy, cultural nuances, and the adaptability of standardized guidelines present formidable challenges. Overcoming these hurdles demands a holistic and context-specific approach that recognizes the intricacies of each setting, ensuring that the benefits of partograph utilization extend across diverse landscapes.

## 10. Cost-benefit analysis of partograph implementation in maternal healthcare

Delving into the economic dimensions of partograph implementation in maternal healthcare unveils a complex interplay of costs and benefits, where investments in this tool yield financial returns and profound improvements in maternal and neonatal outcomes. The narratives from diverse global settings shed light on the multifaceted nature of the cost-benefit analysis associated with partograph utilization.

In a resource-limited district in Uganda, where partograph implementation showcased substantial reductions in maternal complications, the economic analysis revealed a noteworthy return on investment. The upfront costs associated with training programs, monitoring equipment, and partograph forms were outweighed by the significant decrease in the incidence of prolonged labor, emergency interventions, and subsequent healthcare expenditures. The economic analysis underscored the financial sustainability of partograph implementation and highlighted the broader societal gains regarding improved health outcomes and reduced long-term healthcare costs.^[1]^

A contrasting scenario unfolded in a high-income urban setting in Singapore, where the upfront investment in state-of-the-art electronic pantograph systems incurred considerable costs. However, the meticulous economic evaluation demonstrated substantial savings over time. The streamlined data management, reduced instances of adverse events, and optimized resource allocation resulted in long-term cost efficiencies. The economic analysis reinforced that while initial investments might seem substantial, the dividends in improved efficiency and patient outcomes contribute to a compelling cost-benefit equation.^[2]^

The rural landscapes of Nepal offered a unique perspective on the cost-benefit dynamics of partograph utilization. Here, the economic analysis accounted for direct healthcare expenditures and the societal impact of improved maternal health on productivity. Reducing maternal morbidity translated into diminished healthcare costs and increased workforce productivity as healthier mothers contributed more actively to their communities. This holistic approach to cost-benefit analysis positioned partograph implementation as an investment with far-reaching societal implications.^[3]^

The cost-benefit analysis extended beyond healthcare expenditures in a middle-income country like Mexico, where partograph utilization demonstrated a decrease in cesarean section rates and neonatal complications. The economic evaluation factored in the long-term implications of reduced neonatal intensive care unit admissions, improved maternal well-being, and the associated societal gains. This comprehensive analysis emphasized that the benefits of partograph implementation radiate beyond the immediate healthcare setting, contributing to broader societal and economic well-being.^[4]^

The economic narratives converge on a common theme—substantial financial and societal returns counterbalance the upfront costs of partograph implementation. The cost-benefit equation extends beyond healthcare budgets to encompass improved maternal health, reduced long-term healthcare costs, enhanced societal productivity, and a healthier and more resilient community.

## 11. Gender dynamics in maternal healthcare and partograph utilization

e context of partograph utilization unveils a multifaceted narrative shaped by societal norms, power structures, and the pivotal role of women in the maternal health journey.

In rural communities of Bangladesh, where traditional gender roles influence healthcare decision-making, the utilization of partographs reflects an intricate interplay of power dynamics. While women are often the primary recipients of maternal care, the decision to employ partographs is frequently influenced by male family members or community elders. Addressing gender dynamics in partograph utilization involves recognizing and engaging with the broader community, fostering a collective understanding of the tool’s significance and its role in enhancing maternal health outcomes.^[1]^

In contrast, urban settings in Sweden showcase a more egalitarian approach to maternal healthcare and partograph utilization. The societal emphasis on gender equality is mirrored in healthcare practices, where women actively participate in decision-making. Using partographs becomes a shared responsibility, fostering a sense of empowerment and collaboration between healthcare providers and expectant mothers. This gender-inclusive approach contributes to a more holistic and patient-centered maternal healthcare experience.^[2]^

In the diverse cultural landscape of Nigeria, gender dynamics intersect with traditional beliefs and practices surrounding childbirth. Maternal healthcare decisions, including using partographs, are often embedded within familial and communal structures. Recognizing the influence of gender norms requires a tailored approach that involves community leaders, traditional birth attendants, and male family members in educational programs. By fostering a collective understanding, gender dynamics can be navigated to ensure the effective integration of partographs in maternal healthcare.^[3]^

Exploring gender dynamics in a tribal community in India reveals the pivotal role of women as primary caregivers and decision-makers in maternal health. Leveraging this power dynamic, interventions promoting partograph utilization involve community-based women’s groups. These groups serve as conduits for disseminating information and empowering women to advocate for their healthcare needs, challenging traditional gender norms, and fostering a more inclusive approach to maternal healthcare.^[4]^

The gender dynamics in maternal healthcare extend beyond cultural contexts to encompass the technological landscape. In technologically advanced regions like Japan, where electronic partographs are seamlessly integrated into healthcare systems, the gender dynamics of technology access become apparent. Ensuring equitable access and digital literacy among diverse demographic groups are essential in leveraging technology to enhance maternal healthcare outcomes. Addressing gender disparities in technology utilization becomes critical to ensuring inclusivity in partograph implementation.^[5]^

The narratives converge on the recognition that gender dynamics play a pivotal role in shaping maternal healthcare practices, including the utilization of partographs. Tailoring interventions to navigate societal norms, engage diverse stakeholders, and foster inclusivity becomes paramount in ensuring that partograph utilization aligns with women’s needs, expectations, and empowerment in diverse global contexts.

## 12. Monitoring and evaluation framework for partograph utilization

Establishing a robust monitoring and evaluation (M&E) framework for partograph utilization is imperative for assessing its impact, identifying areas for improvement, and ensuring sustained effectiveness within maternal healthcare systems. The narratives from diverse global contexts illuminate the intricacies of M&E frameworks tailored to the dynamic nature of partograph implementation.

In a rural district of Malawi, where partograph utilization has shown promising outcomes in reducing maternal morbidity, the M&E framework encompasses a comprehensive set of indicators. Beyond basic utilization rates, the framework incorporates metrics related to the accuracy of partograph completion, timely interventions, and healthcare provider adherence to protocols. Regular audits and feedback loops are integral components, fostering a culture of CQI and ensuring that the benefits of partograph utilization are consistently realized.^[1]^

Conversely, in an urban tertiary hospital in the Philippines, the M&E framework leverages technology to enhance real-time tracking and analysis. Electronic health record systems integrate partograph data, enabling instant feedback to healthcare providers. Key performance indicators include the frequency of partograph utilization, adherence to evidence-based guidelines, and the correlation between partograph data and maternal outcomes. The technological integration streamlines data management and facilitates evidence-driven decision-making within the healthcare setting.^[2]^

In a community-led initiative in Ethiopia, where partograph utilization is intertwined with local cultural practices, the M&E framework takes a participatory approach. Community members actively engage in the evaluation process, providing feedback on the cultural appropriateness and acceptance of partographs. Qualitative data, including community narratives and perceptions, supplement quantitative metrics. This participatory M&E model fosters community ownership and ensures that partograph utilization aligns with cultural norms, enhancing sustainability.^[3]^

The M&E framework in a regional healthcare network in Brazil emphasizes a multi-stakeholder approach. Beyond traditional healthcare metrics, the framework includes feedback from healthcare providers, expectant mothers, and community leaders. Regular forums for collaborative discussions facilitate a holistic assessment of partograph utilization. This inclusive approach ensures that diverse perspectives are considered, aligning the M&E framework with the varied stakeholders involved in the maternal healthcare journey.^[4]^

A national policy initiative in South Africa showcases the importance of scalability in the M&E framework. Indicators span different healthcare system levels, from primary care clinics to tertiary hospitals. The framework incorporates standardized metrics while allowing flexibility for adaptation to diverse healthcare settings. Regular reporting mechanisms and data sharing between healthcare system levels contribute to a cohesive M&E strategy that aligns with the national policy goals for partograph utilization.^[5]^

These narratives collectively underscore the dynamic nature of M&E frameworks for partograph utilization. From community engagement and technological integration to scalability and inclusivity, effective M&E frameworks go beyond basic utilization rates, delving into the nuanced factors that shape the success of partograph implementation within maternal healthcare systems.

## 13. Sustainability strategies for partograph utilization in maternal healthcare

The sustainability of partograph utilization in maternal healthcare hinges on multifaceted strategies that extend beyond initial implementation, fostering a lasting impact on healthcare systems. The narratives from diverse global contexts offer insights into innovative approaches and enduring strategies contributing to the sustained integration of partographs in maternal healthcare.

In a rural region of India, where partograph utilization faces challenges related to resource constraints, a community-driven sustainability strategy unfolds. Empowering local healthcare workers, especially midwives and community health workers, through continuous training and mentorship programs ensures a skilled and motivated workforce. Integrating partograph training into broader maternal healthcare initiatives embeds the tool within routine practices, contributing to sustained utilization.^[1]^

Conversely, in a highly urbanized healthcare system in the United Kingdom, the sustainability of partograph utilization is woven into a framework of continuous professional development. Regular training sessions, case-based learning, and simulation exercises are integral components. This strategy ensures that healthcare providers remain proficient in partograph interpretation and fosters a culture of adaptability, where the tool evolves in response to emerging best practices and clinical guidelines.^[2]^

Advocacy and awareness campaigns serve as linchpins in a community-led initiative in Nigeria, where cultural acceptance is pivotal for sustained partograph utilization. Community leaders actively disseminate information, dispel myths, and promote the benefits of partograph utilization. This grassroots approach fosters a sense of ownership and cultural integration, contributing to the community’s sustained acceptance and utilization of partographs.^[3]^

The technological landscape in Sweden showcases a sustainability strategy that leverages EHRs. The seamless integration of partographs into EHR systems ensures standardized documentation and accessibility. Regular updates and technological advancements are incorporated to align with evolving healthcare needs. This technological infrastructure enhances the efficiency of partograph utilization and contributes to its sustained integration within the broader healthcare ecosystem.^[4]^

In a national policy initiative in Brazil, where partograph utilization aligns with overarching maternal health goals, sustainability is fostered through policy integration and institutional support. Clear guidelines within national healthcare policies mandate partographs, ensuring a standardized approach across healthcare facilities. Institutional support, including regular audits and feedback mechanisms, reinforces accountability and adherence to partograph protocols, contributing to its sustained utilization at a national level.^[5]^

These narratives collectively emphasize that sustainability strategies for partograph utilization are dynamic and context-specific. From community empowerment and continuous professional development to technological integration and policy alignment, these strategies converge on creating an ecosystem where partographs are implemented and ingrained as indispensable tools in ensuring optimal maternal healthcare outcomes.

## 14. Recommendations

As the tapestry of partograph utilization unfolds within the complex landscape of maternal healthcare, a set of nuanced recommendations emerges, informed by the diverse experiences and strategies discussed.

*Context-specific training programs:* Tailoring training programs to the specific needs and challenges of each healthcare setting is paramount. A one-size-fits-all approach is insufficient for rural communities in sub-Saharan Africa to more than ban tertiary hospitals at the level required. Training initiatives should encompass didactic sessions, practical simulations, and ongoing mentorship to ensure healthcare providers have the skills necessary for effective partograph utilization.^[1]^

*Community engagement and cultural sensitivity*: Recognizing the influence of cultural norms and community dynamics is critical. Advocacy and awareness campaigns should involve community leaders, traditional birth attendants, and women’s groups. These initiatives should be culturally sensitive, dispel myths, and emphasize the benefits of partograph utilization in a manner that resonates with local beliefs and practices.^[2]^

*Technology integration with flexibility*: Leveraging technology, such as electronic partographs and telehealth platforms, enhances accessibility and data management. However, the integration should be flexible, considering varying levels of technological infrastructure. Solutions should be scalable and adaptable to resource-limited and technologically advanced healthcare settings, ensuring inclusivity.^[3]^

*CQI frameworks*: Embracing a culture of constant improvement is essential. Regular audits, feedback loops, and case-based discussions contribute to ongoing learning and refinement of partograph utilization. Quality improvement initiatives should be embedded within healthcare systems, fostering a dynamic approach that evolves in response to emerging best practices and challenges.^[4]^

*Policy alignment and institutional support*: National policies should unequivocally mandate the use of partographs within maternal healthcare protocols. Institutional support, including regular evaluations, accountability mechanisms, and incentives for adherence, reinforces the integration of partographs. Policies should balance standardization and flexibility to accommodate diverse healthcare settings.^[5]^

*Research and innovation*: Encouraging research endeavors that explore the evolving landscape of partograph utilization is vital. Innovation in AI applications, mobile health solutions, or novel training approaches should be supported. Research findings should inform evidence-based updates to guidelines and contribute to the continuous improvement of partograph practices.^[6]^

*Global collaboration and knowledge sharing*: Establishing platforms for worldwide collaboration and knowledge sharing fosters a collective commitment to advancing maternal healthcare. Initiatives should facilitate the exchange of best practices, lessons learned, and research findings. By creating a shared knowledge repository, the international community can collectively overcome challenges and drive innovation in partograph utilization.^[7]^

These recommendations underscore the need for a holistic and adaptive approach to partograph utilization. By addressing training needs, embracing cultural diversity, leveraging technology judiciously, fostering continuous improvement, aligning policies, supporting research, and promoting global collaboration, the international community can navigate the intricacies of maternal healthcare, ensuring that partographs contribute meaningfully to safe and optimal outcomes for mothers and infants.

## 15. Future directions and research priorities in partograph utilization for maternal healthcare

A forward-looking perspective illuminates future directions as partograph utilization continues to evolve within maternal healthcare. It identifies key research priorities to propel the field into new frontiers.

*Integration of AI*: The marriage of partographs with AI holds immense potential. Research should explore AI applications for real-time data analysis, predictive modeling of labor progression, and automated decision support systems. Integrating AI can enhance the accuracy and efficiency of partograph interpretation, providing valuable insights for timely interventions and personalized maternal care.^[1]^

*mHealth solutions*: The ubiquitous presence of mobile devices offers an avenue for innovative interventions. Future research should focus on developing user-friendly mHealth applications that facilitate partograph utilization in diverse settings. These solutions could enhance accessibility, enable remote monitoring, and empower healthcare providers and expectant mothers to engage in the maternal healthcare journey actively.^[2]^

*Maternal health equity*: Understanding and addressing disparities in partograph utilization is crucial. Research should investigate factors contributing to inequities, such as socioeconomic status, geographic location, and cultural norms. Future interventions should aim to design inclusive strategies that bridge these gaps, ensuring that the benefits of partograph utilization are accessible to all women, regardless of their demographic or cultural context.^[3]^

*Longitudinal impact assessment*: Research should extend beyond short-term outcomes to comprehensively assess the longitudinal impact of sustained partograph utilization. Longitudinal studies can elucidate the enduring benefits of maternal and neonatal health, healthcare system efficiencies, and societal outcomes. This research is vital for substantiating the long-term value and sustainability of integrating partographs into routine maternal care.^[4]^

*Adaptability to diverse healthcare systems*: As partograph utilization spans across varied healthcare settings globally, research should focus on developing adaptable models that cater to diverse infrastructures. Future interventions and guidelines should consider the scalability and flexibility of partograph implementation, recognizing the nuances of healthcare systems in both resource-limited and technologically advanced environments.^[5]^

*Patient-centered approaches*: Research endeavors should prioritize understanding the perspectives and preferences of expectant mothers regarding partograph utilization. Investigating the impact of patient-centered approaches, such as shared decision-making and tailored educational interventions, can enhance maternal engagement and satisfaction. Future directions should explore strategies that empower women as active participants in their maternal healthcare journey.^[6]^

*Global collaborative networks*: Establishing collaborative research networks can foster a collective approach to advancing partograph utilization. International collaborations can facilitate the exchange of knowledge, methodologies, and best practices. Future research priorities should include the creation of a global research consortium to address shared challenges, accelerate innovation, and harmonize research efforts on a larger scale.^[7]^

*Cost-effectiveness analysis*: Rigorous economic evaluations should explore the cost-effectiveness of partograph utilization in diverse settings. Research should assess not only the financial aspects but also the broader societal and health system implications. Understanding the economic value of partographs can inform policy decisions and resource allocation, contributing to sustainable implementation strategies.^[8]^

*Psychosocial aspects of maternal care*: Research should delve into the psychosocial dimensions of partograph utilization, considering expectant mothers’ emotional and mental well-being. Exploring the impact of partographs on maternal stress, anxiety, and satisfaction can provide valuable insights for designing holistic interventions that prioritize the overall well-being of women during childbirth.^[9]^

*Innovative training modalities*: Future research should explore innovative training modalities to enhance the proficiency of healthcare providers in partograph utilization. Simulation-based training, virtual reality applications, and interactive e-learning platforms can offer dynamic and engaging educational experiences. Research should assess the effectiveness of these modalities in improving skills, knowledge retention, and real-world application.^[10]^

These future directions and research priorities collectively contribute to the evolution of partograph utilization in maternal healthcare. By embracing technology, promoting equity, assessing long-term impacts, adapting to diverse healthcare systems, prioritizing patient-centered approaches, fostering global collaborations, evaluating cost-effectiveness, exploring psychosocial aspects, and innovating training methodologies, the field can continue to advance, ensuring that partographs play a central role in enhancing maternal and neonatal outcomes worldwide.

## 16. Conclusion

The comprehensive review of partograph utilization in maternal healthcare in Nigeria underscores its pivotal role in enhancing the quality of obstetric care and improving maternal and neonatal outcomes. The journey from historical adoption to contemporary challenges has revealed the multifaceted nature of implementing this essential tool.

While acknowledging the progress made in integrating partographs into national guidelines, there are persistent barriers, including inadequate training, resource limitations, and variability in implementation. The identified incentives, such as improved decision-making and reduced maternal complications, highlight the potential benefits of sustained partograph use.

Global best practices and case studies from other countries have provided valuable insights, emphasizing the importance of standardized protocols, technology integration, and community engagement. The historical context of partograph utilization in Nigeria reflects the evolving landscape of maternal healthcare, with ongoing efforts to address challenges and leverage technological advancements.

Exploring barriers to partograph use in Nigeria highlighted issues such as resistance to change and inadequate resources. On the flip side, incentives for partograph use, including enhanced communication and decision-making, present opportunities for improvement. Government policies and initiatives play a crucial role in shaping the landscape of partograph utilization, requiring strategic interventions for effective implementation.

Case studies provided a nuanced understanding of how diverse settings navigate the challenges and capitalize on incentives for partograph use. Examining recommendations, the emphasis on capacity-building, technology integration, and community involvement emerges as critical pillars for advancing partograph utilization.

The methodology section elucidates the importance of robust data collection and analysis to inform evidence-based practices. As the article explores various headings, including historical context, global best practices, challenges, incentives, and recommendations, the overarching theme revolves around a holistic, multidimensional approach to enhance partograph utilization.

Looking ahead, sustainability strategies encompassing policy integration, continuous training, community engagement, and technological advancements are imperative for embedding partograph utilization into routine maternal healthcare practices. Future research should prioritize AI integration, effectiveness in low-resource settings, and patient-centered approaches to refine further and innovate partograph utilization.

In navigating these complexities, the article serves as a call to action for policymakers, healthcare providers, communities, and researchers to collaboratively work towards ensuring that the use of partographs becomes not just a standard practice but a transformative force in safeguarding the well-being of mothers and newborns in Nigeria and beyond. Through collective efforts and a commitment to evidence-based practices, the vision of comprehensive and effective partograph utilization can be realized, contributing to a brighter, healthier future for maternal healthcare.

## Acknowledgments

We extend our heartfelt gratitude to all those who have contributed to the successful completion of this research on the barriers and incentives influencing the use of partograph in Nigeria. Their support, expertise, and encouragement have been instrumental in shaping the outcomes of this study.

## Author contributions

**Conceptualization:** Chukwuka Elendu.

**Data curation:** Chukwuka Elendu, George Davidson.

**Formal analysis:** Chukwuka Elendu, George Davidson.

**Funding acquisition:** Chukwuka Elendu, George Davidson, John N. Wali, Paschal C. Opara.

**Investigation:** Chukwuka Elendu, George Davidson, John N. Wali, Ucheawaji S. Eneyo, Paschal C. Opara, Prince N. Davidson.

**Methodology:** Chukwuka Elendu, George Davidson, John N. Wali, Philip E. Ebosie, Paschal C. Opara, Prince N. Davidson, Darlington U. Davidson, Junior Davidson.

**Project administration:** Chukwuka Elendu, George Davidson, John N. Wali, Godstime U. Sampson, Ucheawaji S. Eneyo, Philip E. Ebosie, Paschal C. Opara, Prince N. Davidson, Darlington U. Davidson, Junior Davidson.

**Resources:** Chukwuka Elendu, George Davidson, John N. Wali, Godstime U. Sampson, Ucheawaji S. Eneyo, Philip E. Ebosie, Paschal C. Opara, Prince N. Davidson, Darlington U. Davidson, Junior Davidson.

**Software:** Chukwuka Elendu, George Davidson, John N. Wali, Godstime U. Sampson, Ucheawaji S. Eneyo, Philip E. Ebosie, Paschal C. Opara, Prince N. Davidson, Darlington U. Davidson, Junior Davidson.

**Supervision:** Chukwuka Elendu, George Davidson, John N. Wali, Godstime U. Sampson, Ucheawaji S. Eneyo, Philip E. Ebosie, Paschal C. Opara, Prince N. Davidson, Darlington U. Davidson, Junior Davidson.

**Validation:** Chukwuka Elendu, George Davidson, John N. Wali, Godstime U. Sampson, Ucheawaji S. Eneyo, Philip E. Ebosie, Paschal C. Opara, Prince N. Davidson, Darlington U. Davidson, Junior Davidson.

**Visualization:** Chukwuka Elendu, George Davidson, John N. Wali, Godstime U. Sampson, Ucheawaji S. Eneyo, Philip E. Ebosie, Paschal C. Opara, Prince N. Davidson, Darlington U. Davidson, Junior Davidson.

**Writing – original draft:** Chukwuka Elendu, George Davidson, John N. Wali, Godstime U. Sampson, Ucheawaji S. Eneyo, Philip E. Ebosie, Paschal C. Opara, Prince N. Davidson, Darlington U. Davidson, Junior Davidson.

**Writing – review & editing:** Chukwuka Elendu.
